# A low-cost 3D printed resin cell for multimodal X-ray characterization of bio-cementation between two particles

**DOI:** 10.1107/S1600577526009914

**Published:** 2026-10-29

**Authors:** Marilyn Sarkis, Carsten Detlefs, Michela La Bella, Antoine Naillon, Christian Geindreau, Fabrice Emeriault, Yves Watier, James A. D. Ball, Can Yildirim

**Affiliations:** a European Synchrotron Radiation Facility (ESRF) 71 Avenue des Martyrs 38000Grenoble France; b Department of Physics Technical University of Denmark 2800Kongens Lyngby Denmark; c Univ. Grenoble Alpes, CNRS, Grenoble INP, 3SR, F-38000Grenoble, France; ESRF – The European Synchrotron, France

**Keywords:** flow cell, bio-cementation, calcite, S3DXRD, DFXM

## Abstract

We present the design and operation of a low-cost 3D printed resin flow cell for the study of bio-cementation between two aligned particles in contact. The cell supports multimodal synchrotron X-ray characterization of the same interparticle contact, combining 3D X-ray diffraction modalities including scanning 3D X-ray diffraction, X-ray computed tomography and darkfield X-ray microscopy across multiple beamlines at the European Synchrotron Radiation Facility. Its geometry allows calcite crystals precipitated at the particle contact to be unambiguously distinguished from those nucleated on the cell walls during both indexing and crystal selection. The platform is demonstrated on glass beads and S30 sand grains cemented over successive cycles under varying NaCl concentrations, resolving the morphology and orientation of individual calcite crystals from the grain scale down to the intragranular scale. Practical limitations of the current design are also discussed.

## Abstract 2

A novel, low-cost 3D printed resin flow cell is presented, in which two particles can be placed in contact at a synchrotron beamline for interrupted *in situ* measurements.

## 1. Introduction

Bio-cementation is a process that can be used to reinforce loose soils such as sand, and to fabricate construction materials. It relies on bacterial activity, primarily that of *Sporosarcina pasteurii*, which hydrolyses urea present in the environment (Ehrlich, 1999 ▸; Ferris *et al.*, 1996 ▸; Castanier *et al.*, 2000 ▸). In the presence of a source of calcium cations (Ca^2+^), calcite crystals (CaCO_3_) precipitate in the matrix, and act as a binder between the sand particles, analogous to cement. Typically, the cementation can be done in cycles. The more cycles performed on the soil, the higher the amount of CaCO_3_ precipitated.

The mechanical behavior of the composite material has been extensively studied in the literature, but the characterization of the *3D microstructure* of the precipitated calcite crystals using diffraction imaging techniques such as scanning 3D X-ray diffraction (S3DXRD), 3D X-ray diffraction (3DXRD) and darkfield X-ray microscopy (DFXM) is only very recent work (La Bella *et al.*, 2025 ▸; Sarkis *et al.*, 2026 ▸). Bio-cementation being an inherently dynamic process, the ultimate goal is to capture *in situ*, across successive cementation cycles, how the microstructure and internal deformation of the precipitated crystals govern the mechanical response of the overall material. The aforementioned studies, however, did not vary the different conditions under which these crystals can precipitate. Addressing this requires well controlled environments capable of imposing specific cementation conditions, such as reactant concentrations and the nature of the substrates on which crystals nucleate and grow, while remaining compatible with the constraints of the characterization techniques employed. In the case of X-ray diffraction-based methods, the sample environment must exhibit sufficient transmission, withstand beam-induced heating, and minimize background contributions to the acquired data. For this, 3D resin printing presents itself as an economical and effective solution (Perera *et al.*, 2026 ▸).

This paper presents a novel, low-cost 3D printed resin flow cell, in which two particles can be placed in contact at a synchrotron beamline for interrupted *in situ* measurements. The reactants necessary for bio-cementation to take place can be injected, and bio-cementation will occur. In this work, we present a low-cost, 3D-printed resin flow cell designed to perform bio-cementation between two aligned particles in contact and to enable multimodal synchrotron X-ray characterization of the resulting calcite microstructure between successive cementation cycles. Two types of substrate, glass beads and sand grains, are investigated, and the salinity of the medium is varied to probe its influence on the precipitated crystals. The cell design is first described, followed by the experimental set-ups and measurements carried out at the European Synchrotron Radiation Facility (ESRF): S3DXRD and X-ray computed tomography (XCT) on ID11, and DFXM on ID03.

## 2. The flow cell

**Figure 1:**
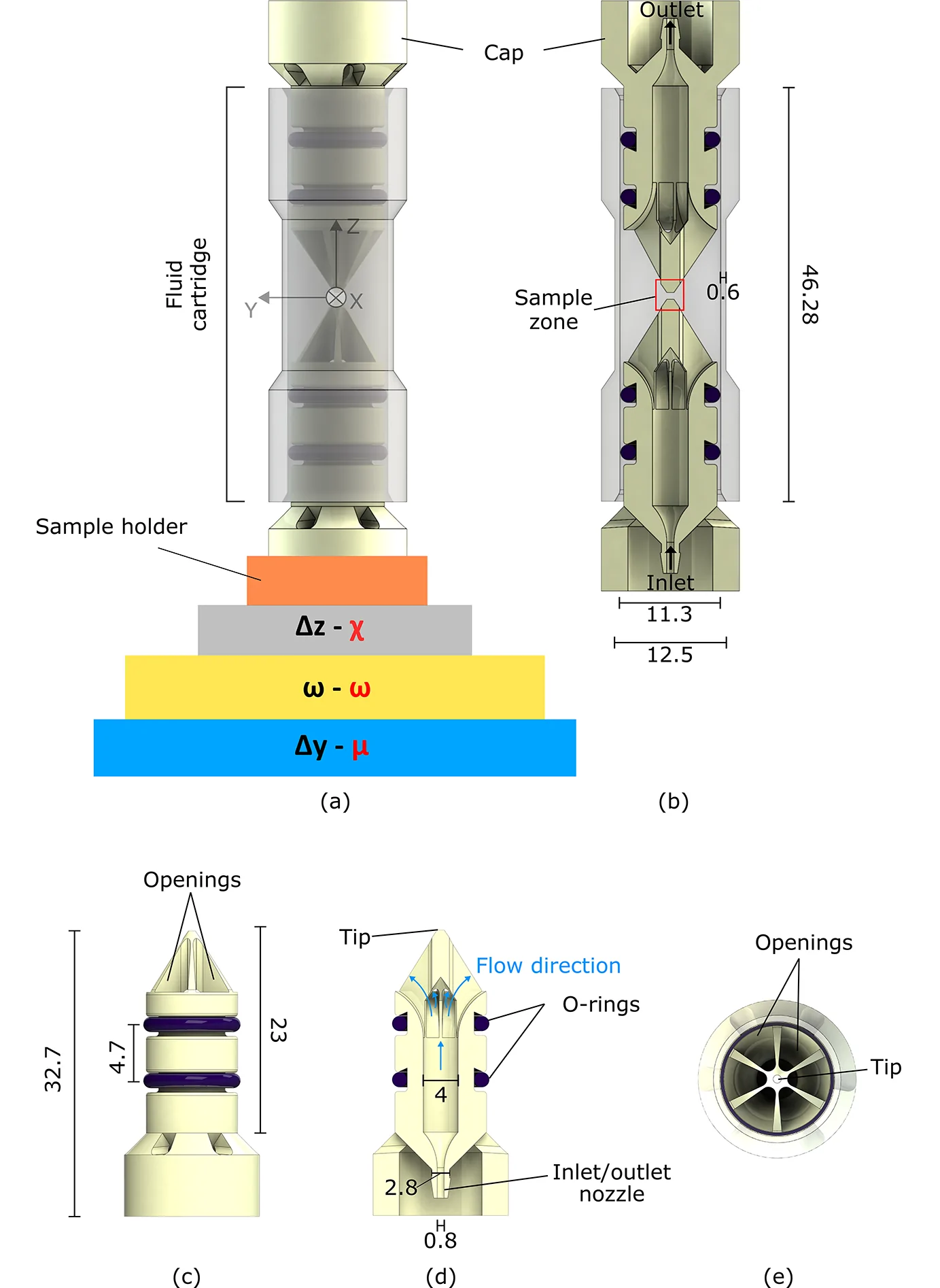
(*a*) A 3D render of the whole flow cell, with the motor stacking at both ID11 (in black; Δ*y*: translation along the laboratory *Y*-axis; ω: rotation around the laboratory *Z*-axis; Δ*z*: translation along the laboratory *Z*-axis) and ID03 (in red; μ: rocking angle around the laboratory *Y*-axis; ω: rotation around the laboratory *Z*-axis; χ: rotation around the laboratory *X*-axis). (*b*) A vertical section of the cell. (*c*) 3D render of one cap. (*d*) Vertical cross-section of the cap in (*c*). (*e*) Top view of the cap showing the six equally spaced openings for the fluid circulation. All dimensions are in mm.

The flow cell presented in this work is a 3D SLA-printed device designed to bring two particles into contact and to enable bio-cementation by circulating the required fluids around them. It was printed using the Form 3 printer from Formlabs, using Clear Resin V4.1 (Formlabs, 2024 ▸). As detailed in the following sections, the cell is primarily intended for S3DXRD and XCT at 43 keV on ID11 (Wright *et al.*, 2010 ▸), and has also been used for DFXM at 17 keV on ID03 (Isern *et al.*, 2025 ▸). The cell is made up of two parts:

(i) *The end caps*. Two caps are needed for each cell, placed at each end of the fluid cartridge, as per the corresponding drawings in Figs. 1 ▸(*a*) and 1(*b*). Each cap has a nozzle with a diameter of 0.8 mm that can connect it to a flexible tube, such that one end is the inlet of the cell and the other is the outlet, as shown in Fig. 1 ▸(*b*). At the flat tip of each cap, a particle—such as a sand grain or glass bead—can be glued. Two greased O-joints around each cap are needed, such that it slides straight through the hollow cylinder, and the two particles can be therefore aligned inside the cell and put in contact. The flat tip is atop a conical shape with six equally spaced areas around the radius to allow the fluids to circulate from the nozzle to the inside of the cell [Fig. 1 ▸(*e*)]. Once the cell is closed, the two particles in contact should sit at mid-height of the cell—approximately 33 mm from the far edge of one of the two caps, as depicted in Fig. 1 ▸(*b*). This distance varies with the size of the two particles that are put in contact.

(ii) *The fluid cartridge*. A hollow cylinder, of inner diameter 11.3 mm, outer diameter 12.5 mm and height 46.28 mm, as shown in Figs. 1 ▸(*c*), 1(*d*) and 1(*e*).

A critical step in the use of the cell is the alignment of the two particles in contact, which must be verified before any bio-cementation cycle. This is not straightforward: although the SLA resin is nominally transparent, the as-printed surface of the cartridge is too rough to allow optical inspection of its interior. To address this, the fluid cartridge is polished with sandpapers of decreasing grain size (down to 0.3 µm). Once wet with water, the cylinder becomes transparent enough for the contact between the two particles to be checked under an optical microscope. The cell is then sealed with optical glue (NOA81) to immobilize the caps and preserve the contact, the same glue being used to fix each particle to its cap tip.

In this paper, we report on the interrupted *in situ* X-ray measurements, in order to avoid tubing during the full 360° rotations. The flow rate was set to 63.84 µL min^−1^, which is the suitable value for bio-cementation. This flow rate was applied using a syringe pump, which also allowed the fluids to be withdrawn from the cell before performing the measurements. A dedicated 3D printed holder was used for the measurements on ID11 and ID03, to bring the two particles to the required height at each beamline.

In total, three samples were measured:

(i) Sample A: with two sand grains in contact, and a concentration of NaCl of 3 g L^−1^.

(ii) Sample B: with two glass beads in contact, and a concentration of NaCl of 3 g L^−1^.

(iii) Sample C: with two sand grains in contact, and a concentration of NaCl of 9 g L^−1^.

For all measurements, the cell provided very high transmission. Even though the actual thickness of the wall is smaller than the one in the initial drawings due to the polishing, the theoretical calculations showed around 97% and 87% transmission for 43 and 17 keV, respectively.

## 3. S3DXRD, CT and DFXM measurements

### 3.1. S3DXRD and CT on ID11

At the European Synchrotron Radiation Facility ID11 beamline (Wright *et al.*, 2020 ▸), a monochromatic micro-focused X-ray beam of 800 nm in size with an energy of 43 keV was used for scanning 3DXRD. Fig. 1 ▸(*a*) shows how the sample was mounted atop the beamline motors for S3DXRD. The sample was mounted vertically and rotated 180° with the ω motor around the laboratory *Z*-axis for each Δ*y* translation along the laboratory *Y*-axis in the beam (Henningsson *et al.*, 2020 ▸; Hayashi & Kimura, 2023 ▸; Li *et al.*, 2023 ▸; Zhang *et al.*, 2025 ▸; Kim *et al.*, 2026 ▸). Different layers were scanned by translating the sample vertically through Δ*z*. The sample was mounted such that the long axis of the cell was roughly parallel to the laboratory *Z*-axis. The diffraction patterns were collected using a DECTRIS Eiger2X CdTe 4M detector. In total, three samples were scanned: sample A was prepared using glass beads of around 1 mm in contact, and samples B and C using S30 sand grains (Sarkis *et al.*, 2024 ▸; Sarkis *et al.*, 2025 ▸) in contact of diameter around 0.8 µm. For CT, a box beam of 1 mm × 1 mm was used to illuminate the region of interest. Tomographic images were collected on both the Marana3 detector and a Basler camera with respective pixel sizes of 0.55 µm pixel^−1^ and 0.93 µm pixel^−1^.

The objective of this experiment was to observe the microstructure, shape and orientation of each calcite crystal across different cycles, and under different conditions. The cementation protocol applied was the same one mentioned by Sarkis *et al.* (2024 ▸) and Sarkis *et al.* (2025 ▸): after saturating the cell with water (Crystaline – Sainte Cécile), 2 pore volumes (PV) of the bacterial solution were injected which consisted of 1 g L^−1^ of bacteria and NaCl. After waiting a few hours for the bacteria to attach, 1 PV of the cementing solution [equimolar solution of CaCl_2_ and CO(NH_2_)_2_] was injected, and then repeated 2 h later. These injections consisted of one cementation cycle. Samples A and B had NaCl concentrations of 3 g L^−1^, while sample C had an NaCl concentration of 9 g L^−1^. After each cementation cycle, S3DXRD data were collected in layers around the contact area, to observe the evolution of the calcite crystals under different conditions.

**Figure 2:**
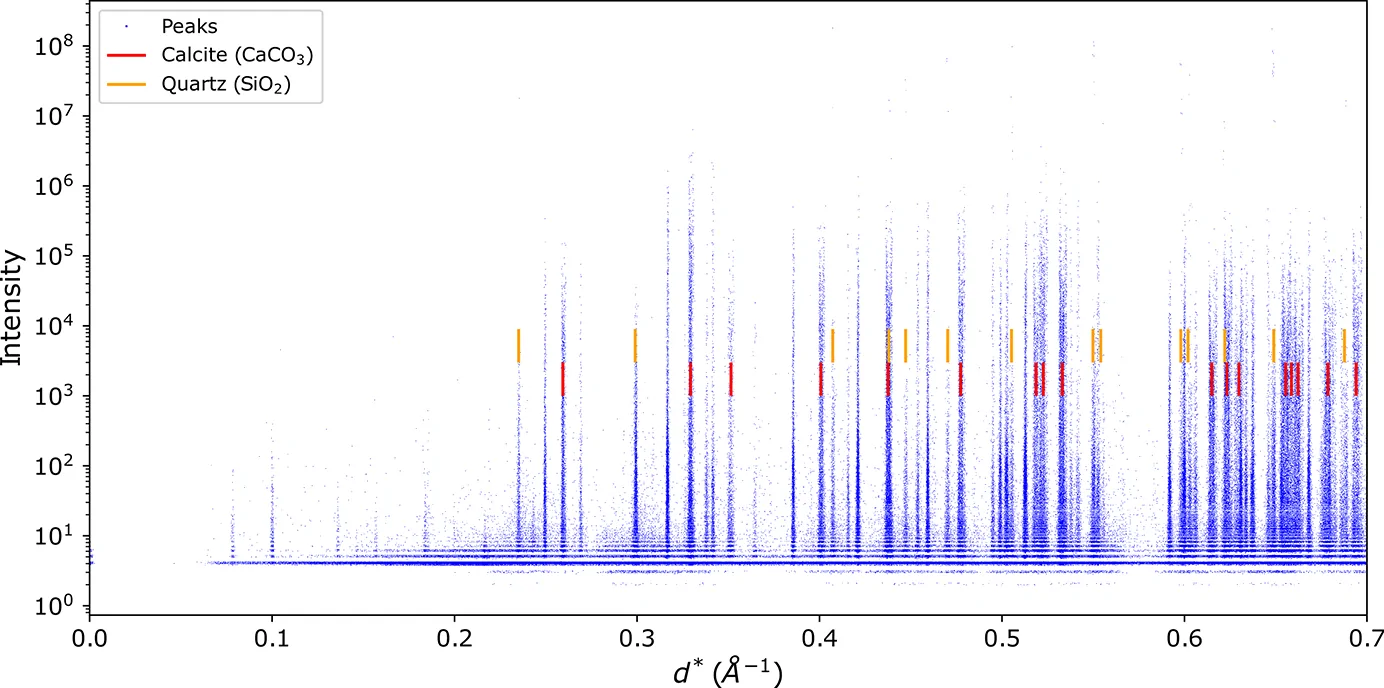
Plot showing the diffracted peak intensity against 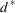, with the rings corresponding to calcite and quartz, for a layer in sample B after the first cementation cycle. The numbers of plotted peaks and rings are reduced for visualization purposes.

Fig. 2 ▸ shows the segmented diffraction peaks for a layer in sample B. Bragg diffraction spots were identified on each detector image by intensity thresholding and segmentation into connected pixel clusters. For each segmented diffraction peak, the integrated intensity and reciprocal lattice spacing (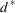) were calculated using the calibrated detector geometry. The diffraction peaks from all detector images acquired during the S3DXRD measurement were then combined into a single dataset. Fig. 2 ▸ displays the integrated intensity of each segmented diffraction peak as a function of 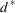. The vertical markers indicate the theoretical Bragg reflection positions of calcite and quartz, allowing phase identification. Only two phases are present, which are the calcite and the quartz, with some additional small-angle scattering coming from the resin cell, at a significantly reduced intensity than the wide-angle diffraction peaks. However, since the calcite can precipitate anywhere in the cell, and not only at the contact between the two particles but also on the cylinder walls, each calcite ring is present three times: twice for each cylinder wall in the beam path and once for the actual contact. Only the crystals present at the contact, *i.e.* with the peaks lying in the middle ring between the three, were considered for indexing, as shown in Fig. 2 ▸. In this plot, 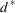 corresponds to the reciprocal space lattice spacing, such that 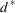 = 1/*d*, where *d* is the lattice spacing in real space.

**Figure 3:**
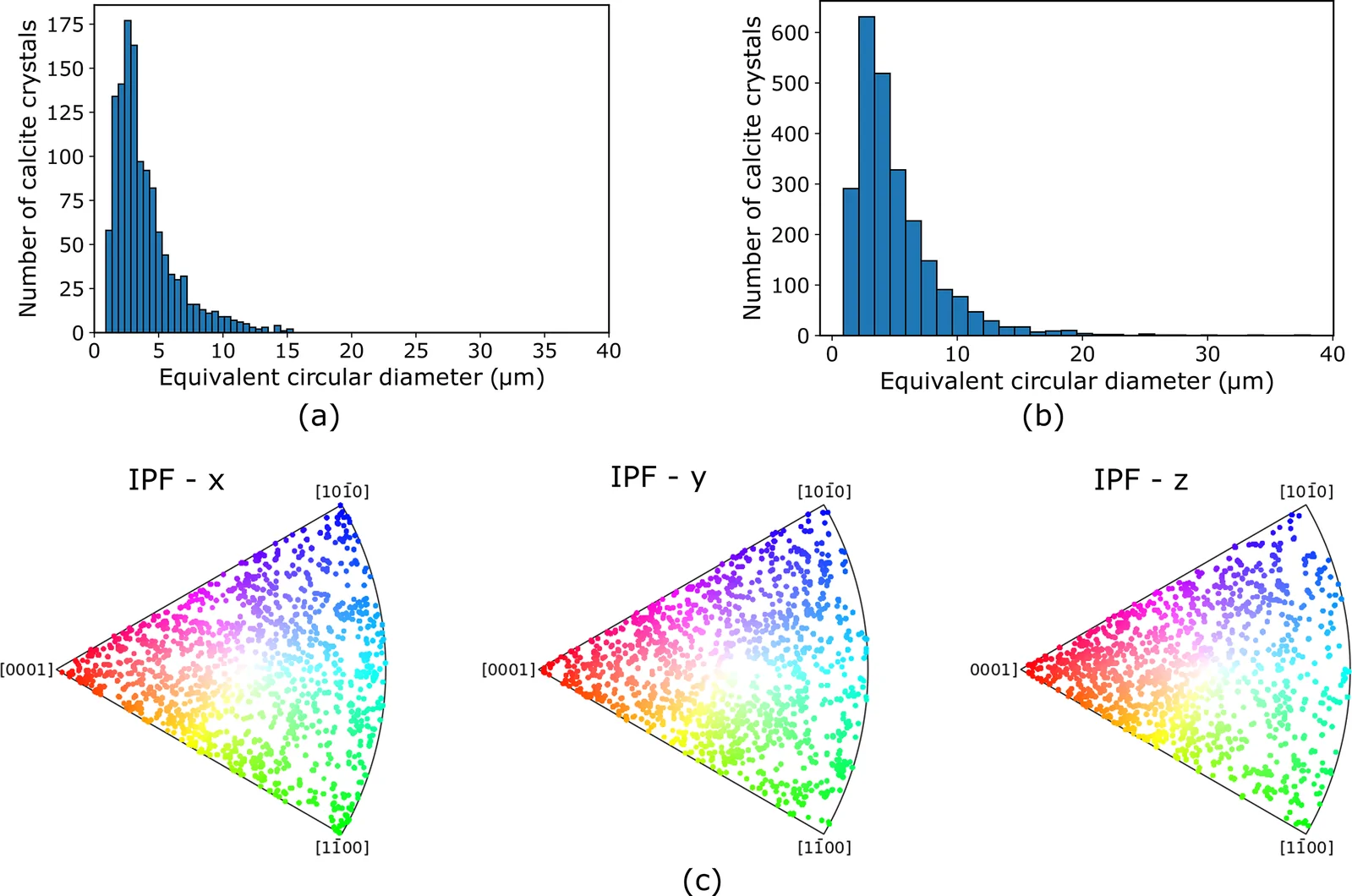
For the sample A, and for the middle layer of the scanned area of the contact, (*a*) histogram of the equivalent circular diameter, ECD, of the calcite crystals after one cycle of biocementation; (*b*) histogram of the ECD of the calcite crystals after three cycles of biocementation, and (*c*) the inverse pole figures, IPFs, along the *x*, *y* and *z* laboratory axes for one cycle of cementation.

Fig. 3 ▸ shows the distribution of the equivalent circular diameter (ECD) of the calcite crystals for the sample with sand grains and a concentration of 3 g L^−1^ of NaCl, only around the sand grains, *i.e.* excluding the crystals that precipitated away from the sand grains close to the walls of the cell. The area distribution is computed for the calcite crystals indexed after one cementation cycle and three cementation cycles. In the first case, 1269 calcite crystals precipitated around the contact with a mean ECD of 3.91 µm, and 2464 crystals in the latter with a mean ECD of 5.08 µm. Fig. 3 ▸(*c*) shows the inverse pole figures (IPFs) for the calcite crystals along the *x*, *y*, *z* laboratory axes. As found in La Bella *et al.* (2025 ▸) and Sarkis *et al.* (2026 ▸), the calcite crystals here present a preferential growth orientation, where most of them grow with the *c*-axis along the laboratory *z*-axis, which is the axis roughly perpendicular to the sand grains.

**Figure 4:**
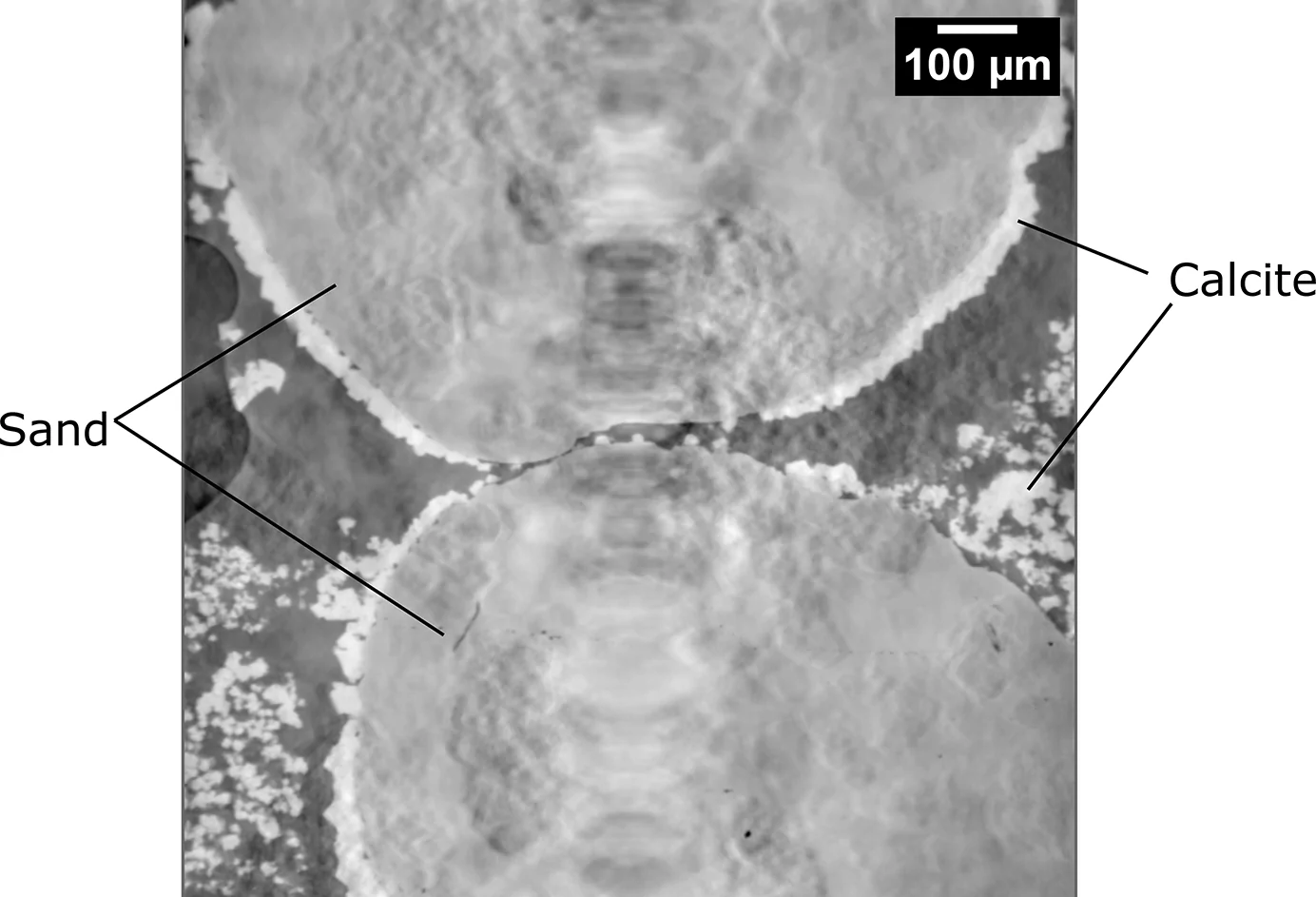
Slice of the 3D volume acquired using computed tomography on the Marana3 detector for sample B after the third cementation cycle.

As for the phase contrast tomography (PCT), the relatively large diameter of the cell did not allow for the cell to be completely out of the beam path while collecting the flatfield images, due to the limited range of the translation motor. For this reason, as shown in Fig. 4 ▸, the reconstructed images show some associated artifacts, which complicate the subsequent data analysis procedures. Proper flatfields can be taken, provided the *y*-translation motor can be translated around 7 mm to get the cell out of the beam path, which was not possible during the experiment. PCT remains an independent technique from S3DXRD, and the size of the cell did not complicate the S3DXRD measurements.

### 3.2. DFXM on ID03

The main technique performed on ID03 is darkfield X-ray microscopy (Isern *et al.*, 2025 ▸). The measurements were performed at 17 keV, which implies more flux and absorption on the material side than at 43 keV. On ID03, two beam modes are possible: (i) the box-beam mode, and (ii) the line-beam mode. In the second mode, the beam is focused with a set of 58 1D lenses downstream of the sample into a more intense beam with a height of around 500 nm, which enables layer measurements on a given crystal (Sarkis *et al.*, 2026 ▸). The setup for DFXM on ID03 is shown in Fig. 1 ▸(*a*). The sample was also mounted vertically such that its long axis was parallel to the laboratory *Z*-axis. The measurements were performed by rotating the sample around the laboratory *Y*-axis using the rocking motor μ, and around the laboratory *X*-axis using the rolling motor χ.

The first challenge was to manage to image a calcite crystal that was at the contact between the two sand grains, and not precipitated on the cell walls. First a nearfield detector is typically used at the Bragg downstream from the sample, then is projected to the farfield detectors. Once aligned, the objective is placed between the sample and the farfield detector along the diffracted signal for the grain and the objective is focused by moving it along the optical axis. Thanks to the large diameter of the cell, once the reflection was found in the nearfield detector and well aligned in the center of rotation of the goniometer at ID03, the sample was rotated in small steps around the laboratory *Z*-axis using ω [Fig. 1 ▸(*a*)]. Thanks to the large diameter of the cell, if the aligned crystal was indeed on the walls, its reflection would disappear. If a reflection remains after a rotation of a few degrees, then the crystal is indeed at the contact between the two particles.

The DFXM measurements performed at 17 keV on ID03 were challenging. When the beam was focused using the 1D lenses to perform layer scans, a slow positional drift of the illuminated crystal was observed, caused by local softening of the resin, without any loss of integrity of the cell, which remained sealed with the particles in contact throughout. The drift was corrected by realignment between layers and during post-processing, and did not prevent the measurements from being carried out. This beam-induced heating issue could be mitigated in future experiments by operating at higher X-ray energies, where absorption in the sample is significantly reduced; diamond compound refractive lenses have recently been demonstrated to be effective at 37 keV in DFXM mode (Staeck *et al.*, 2026 ▸), making this a promising avenue for minimizing thermal damage to radiation-sensitive samples.

## 4. Discussion

The flow cell presented in this work was designed as a versatile sample environment for multimodal synchrotron characterization of reactive interfaces. Unlike sample holders dedicated to a single experimental technique, the proposed design is compatible with tomography, scanning 3DXRD and DFXM, allowing complementary morphological, crystallographic and strain information to be acquired on the same specimen while preserving the region of interest. Furthermore, the cell enables controlled positioning and contact between two individual objects, making it particularly suitable for studying interfacial processes *in situ*.

The use of stereolithography-based 3D printing provides considerable flexibility in the design of the sample environment. The geometry can be readily adapted to accommodate different specimen dimensions, contact configurations or fluidic layouts without modifying the overall concept. This approach also offers a rapid and cost-effective alternative to conventionally machined sample environments, facilitating the fabrication of application-specific designs while maintaining compatibility with synchrotron experiments. For one complete cell, including building supports, 17 ml of Formlabs Clear resin are needed. This makes a total raw material cost of less than 4 Euros per cell. The very low fabrication cost of the cell is another practical advantage of the proposed design. The cells can be treated as consumable components, allowing several cells to be prepared in advance for different experimental conditions and readily replaced in case of damage. This is particularly advantageous for experiments involving long reaction times, such as the cementation cycle investigated here, as the loss of a cell does not significantly affect the available beam time.

The measurements performed in this study also highlight practical considerations associated with polymer-based sample environments. While the selected resin provides adequate X-ray transmission and mechanical stability for the experiments presented here, prolonged exposure to high X-ray fluxes or chemically aggressive environments may require the use of alternative printing materials depending on the experimental conditions. Consequently, the choice of printing material should be adapted according to the beam energy, radiation dose and chemical environment of the intended application.

Moreover, the large diameter of the fluid cartridge was particularly useful for the S3DXRD indexing and the DFXM measurements in order to be able to find a calcite crystal that is precipitated at the contact, thanks to the tripled rings for each reflection. However, the radius of the cell was larger than the movement limit of the *y*-translation motor, which caused the cell to remain in the beam for the flatfields. The presented cell can be very well used for *in situ* experiments as done by Perera *et al.* (2026 ▸), which was not the case for the performed experiments.

Beyond the bio-cementation experiments demonstrated in this work, the modular design can readily be extended to other studies involving controlled interfaces between two objects. Examples include real-time crystal growth monitored by DFXM, calcite dissolution under controlled chemical conditions, operando electrochemical experiments involving two electrodes and electrolyte circulation, and gas–solid reactions such as humidity-controlled mineral growth or dissolution. The fluidic architecture can also be modified by incorporating additional inlet and outlet channels, allowing the sequential or simultaneous delivery of multiple liquid or gaseous reactants. These possible adaptations illustrate the versatility of the proposed platform for a wide range of operando synchrotron investigations.

## 5. Conclusion

A low-cost 3D printed resin flow cell has been designed, fabricated, and demonstrated for multimodal synchrotron X-ray characterization of bio-cementation between two aligned particles in contact. The cell achieves high X-ray transmission at both 17 and 43 keV, is compatible with S3DXRD, XCT, and DFXM on separate beamlines, and provides a straightforward geometric means of distinguishing crystals nucleated at the interparticle contact from those precipitated on the cell walls. Applied to glass beads and S30 sand grains cemented under varying NaCl concentrations, it resolved individual calcite crystal morphologies and orientations from the grain scale down to the intragranular level. Beam-induced heating at 17 keV remains the main limitation for DFXM, but higher-energy operation enabled by diamond CRLs offers a clear mitigation path. The design is readily adaptable to a wide range of contact-controlled experiments beyond bio-cementation, including dissolution, electrochemistry, and gas-phase reactions

## Data Availability

Data will be made available upon request
